# Real-world practices and challenges of radiologically isolated syndrome: results of a cross-sector survey by the DACH MS guidelines group

**DOI:** 10.3389/fneur.2026.1822692

**Published:** 2026-07-14

**Authors:** Friederike Held, Antonios Bayas, Katharina Christe, Christian Dettmers, Burkhard Domurath, Juliane Ebert, Edeltraud Faßhauer, Peter Flachenecker, Jutta Gärtner, Dzenita Hasanbasic, Christoph Heesen, Harald Hegen, Thomas Henze, Peter Huppke, Veronika Kana, Michael Khalil, Ruth Kirschner-Hermanns, Thomas Korn, Tania Kümpfel, Sabine Lamprecht, Uwe Meier, Gerd Meyer zu Horste, Mathias Mäurer, Frederike Cosima Oertel, Caroline Pot, Anne-Katrin Pröbstel, Kevin Rostásy, Anke Salmen, Jutta Scheiderbauer, Markus Schmidt, Erwin Stark, Corinna Trebst, Regina Trollmann, Clemens Warnke, Mike P. Wattjes, Benedikt Wiestler, Brigitte Wildemann, Uwe Zimmermann, Klaus Gehring, Achim Berthele, Bernhard Hemmer

**Affiliations:** 1 Department of Neurology, TUM Hospital, Munich, Germany; 2 Division of Immunology and Rheumatology, Stanford University, Stanford, CA, United States; 3 Department of Neurology, Medical Faculty, University of Augsburg, Augsburg, Germany; 4 EASW Ergotherapy Academy Southwest gGmbH Furtwangen; Hochschule Furtwangen, Furtwangen, Germany; 5 Schmieder Clinics Konstanz, Konstanz, Germany; 6 Beelitz Clinics GmbH, Michendorf, Germany; 7 Valens Clinics, Davos Platz, Switzerland; 8 German MS Society (DMSG), Halle, Germany; 9 Quellenhof Neurological Rehabilitation Center, Bad Wildbad, Germany; 10 University Medical Center Göttingen, Göttingen, Germany; 11 Brandenburg Medical School Theodor Fontane, Neuruppin, Germany; 12 INIMS, University Medical Center Hamburg-Eppendorf, Hamburg, Germany; 13 Medical University of Innsbruck, Innsbruck, Austria; 14 Private, Lappersdorf, Germany; 15 Jena University Hospital, Jena, Germany; 16 Department of Neurology, University Hospital Zurich, Zürich, Switzerland; 17 Medical University of Graz, Graz, Austria; 18 Neuro-Urology/Urology University Clinic of Bonn (ret), Neuro-Urology, Johanniter Neurological Rehabilitation Center Bonn, Göttingen, Germany; 19 Institute for Experimental Neuroimmunology, Munich, Germany; 20 Institute of Clinical Neuroimmunology, LMU University Hospital, LMU Medizin, Ludwig-Maximilians-Universität München, Munich, Germany; 21 HSH Lamprecht Partnership, Kirchheim unter Teck, Germany; 22 NeuroCenter Grevenbroich/Dormagen, Dormagen, Germany; 23 University Hospital Münster, Münster, Germany; 24 Würzburg Mitte Hospital, Würzburg, Germany; 25 Experimental and Clinical Research Center, Charité Berlin, Berlin, Germany; 26 Einstein Center Digital Future, Berlin, Germany; 27 Lausanne University Hospital (CHUV), Lausanne, Switzerland; 28 University Hospital Bonn, Bonn, Germany; 29 German Center for Neurodegenerative Diseases (DZNE), Bonn, Germany; 30 University Hospital Basel, Basel, Switzerland; 31 Research Center for Clinical Neuroimmunology Basel, Basel, Switzerland; 32 Vestische Children's Hospital Datteln, Datteln, Germany; 33 Department of Neurology, St. Josef-Hospital, Ruhr University Bochum, Bochum, Germany; 34 Foundation for Self-Determination MS, Trier, Germany; 35 Sana Clinics Duisburg, Duisburg, Germany; 36 Private, Mühlheim, North Rhine-Westphalia, Germany; 37 Hannover Medical School, Hanover, Germany; 38 Department of Pediatrics, Pediatric Neurology, Friedrich-Alexander-University Erlangen-Nürnberg, Erlangen, Germany; 39 University Hospital Marburg, Marburg, Germany; 40 Charité Berlin, Institute for Neuroradiology, Berlin, Germany; 41 Institute for Diagnostic and Interventional Neuroradiology, TUM Hospital, Munich, Germany; 42 Heidelberg University Hospital, Heidelberg, Germany; 43 University Medicine Greifswald, Greifswald, Germany; 44 Neurocenter at Klosterforst GmbH, Itzehoe, Germany; 45 Munich Cluster for Systems Neurology (SyNergy), Munich, Germany

**Keywords:** clinical guidelines, healthcare survey, multiple sclerosis, off-label treatment, radiologically isolated syndrome

## Abstract

**Objective:**

To evaluate real-world Radiologically isolated syndrome (RIS) management practices across Germany, Austria, and Switzerland and identify key gaps for future guideline development.

**Methods:**

Between October 1 and October 16, 2024, neurologists across university hospitals, teaching hospitals, municipal clinics, and private practices in Germany, Austria, and Switzerland (DACH) participated in an anonymous online survey on RIS diagnosis, treatment, and monitoring practices.

**Results:**

Among 127 physicians from diverse care settings, RIS management showed both marked heterogeneity and notable convergence. While the diagnostic work-up was relatively uniform (>85% performed comprehensive magnetic resonance imaging (MRI) and cerebrospinal fluid (CSF) analysis, as well as evoked potentials), awareness and implementation of the 2023 RIS criteria varied substantially (34% consistent use, 42% partial implementation, 16% non-adoption, and 8% unaware). Treatment practices showed the greatest heterogeneity: although 54% considered therapy initiation and clinicians agreed on treatment triggers (high lesion burden, new MRI activity, inflammatory CSF), 69% treated less than 25% of their RIS cases. Off-label treatment strategies, medication choices, and escalation approaches varied widely across settings, with 72% lacking standardized algorithms. Structural barriers were consistent across all countries and care levels, with reimbursement challenges (61%), patient communication difficulties (42%), and limited access to specialists outside university centers identified as key obstacles. Recent treatment trials increased therapeutic confidence, yet clinicians emphasized the urgent need for national recommendations to harmonize diagnostic workflows, treatment pathways, and reimbursement structures.

**Conclusion:**

Real-world RIS care in the DACH region is characterized by substantial practice heterogeneity, coexisting with shared clinical approaches, which reflects the absence of formal guidelines and approved therapies. While the 2024 McDonald criteria now enable immunotherapy for asymptomatic individuals at high-risk for MS, clinical uncertainty persists regarding risk stratification, treatment decisions, and communication frameworks, challenges applicable to both RIS and preclinical MS. These findings reveal critical unmet needs and demonstrate the urgent necessity for harmonized DACH-wide recommendations addressing diagnostic standards, risk-adapted treatment pathways, patient communication, reimbursement integration, and cross-sector collaboration.

## 1 Introduction

Radiologically Isolated Syndrome (RIS) represents the earliest stage to detect individuals with enhanced risk for multiple sclerosis (MS). Longitudinal trials showed that approximately 50% of individuals with RIS will develop MS symptoms within 10 years, with a further increase to 70% within 20 years of observation time (1). The 2023 RIS criteria enable the earlier diagnosis of individuals with an enhanced risk for MS compared to the 2009 RIS criteria (2, 3). However, these criteria show low specificity, creating difficulties in treatment decisions and patient counseling. Additionally, the revised 2024 McDonald criteria, which allow MS diagnosis in asymptomatic individuals with specific risk factor constellations, create diagnostic overlap (4). While a notable proportion of cases now covered by the McDonald criteria may access approved drugs previously only available off-label, uncertainty remains, given the low specificity of the RIS criteria, and long-term data are lacking. Given that RIS does not describe a specific disease entity and rather a transitional concept characterized by biological, diagnostic, and clinical uncertainty, clinicians face challenging risk–benefit considerations: preventing irreversible CNS injury versus treating asymptomatic individuals. Despite emerging evidence from early treatment trials with dimethyl fumarate (ARISE) and teriflunomide (TERIS) versus placebo, clinical uncertainty persists regarding which cases benefit from early intervention (5, 6). Standardized biomarkers are currently under investigation but clear evidence to reliably predict MS symptom onset is largely lacking (7).

Within the DACH region, regulatory challenges exist as RIS lacks specific ICD codes, diagnostic guidelines, and licensed therapies. Reimbursement strategies influence the availability of cost-intensive diagnostics and disease-modifying treatments, with MS-related services regulated through specific national contracts from which RIS is excluded. Despite different healthcare systems across Germany, Austria, and Switzerland, a region-independent gap in guidelines and national framework conditions persists.

This study addresses the critical knowledge gap in real-world RIS clinical practices across the DACH region. Understanding current practice heterogeneity and urgent needs is essential for developing standardized guidelines.

## 2 Materials and methods

### 2.1 Study design and objectives

This cross-sectional survey study was conducted to assess current real-world practices in the diagnosis, treatment, and monitoring of RIS across the DACH region (Germany, Austria, and Switzerland). The survey was designed to identify areas of consensus and heterogeneity in clinical practice and unmet needs in RIS care.

### 2.2 Survey development

The survey questionnaire was developed by a team of neurologists with expertise in MS care, in collaboration with the DACH MS guideline group. The questionnaire addressed diagnostic approaches, treatment practices, monitoring strategies, communication with patients, and regulatory challenges. It consisted of single-choice (*n* = 8) and multiple-choice (*n* = 9) questions, complemented by free-text fields (*n* = 8) to capture qualitative insights.

### 2.3 Participant recruitment and data collection

Between October 1 and 16, 2024, the MS guideline group invited board-certified neurologists from Germany, Austria, and Switzerland to participate using a snowball sampling (a non-probability recruitment technique in which existing participants recruit further eligible participants from among their colleagues) (8). The survey was answered anonymously using an online platform *(LamaPoll*), with no personally identifiable information collected. Participants were informed about the study’s purpose and data handling procedures; consent was implied by survey completion. The survey remained open until October 16, 2024. A total of 130 neurologists accessed the survey. Three respondents with no completed items were excluded. Of the remaining 127 participants, 110 (86.6%) completed the entire survey, while 17 (13.4%) provided partial responses with a mean completion rate of 89.2%. All 127 respondents were included in the analysis.

### 2.4 Data analysis

Quantitative data were analyzed using descriptive statistics, with categorical variables reported as frequencies and percentages. Responses were stratified by care setting where appropriate. Free-text responses were analyzed using qualitative content analysis by two independent investigators (FH and ABe).

### 2.5 Ethical considerations and reporting standards

The survey was conducted in accordance with the Declaration of Helsinki and national data protection regulations. Given its anonymous nature and focus on professional practices, formal ethical approval was not required. This study was reported in accordance with the Checklist for Reporting Results of Internet E-Surveys (CHERRIES) (9).

## 3 Results

### 3.1 Participants and center characteristics

**Figure 1:**
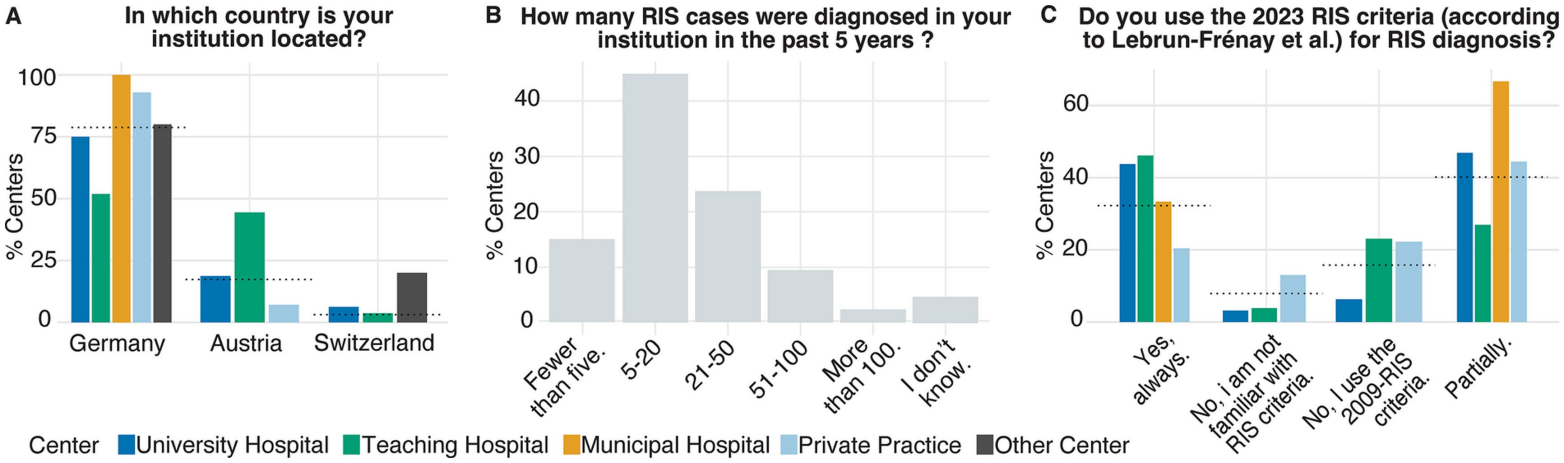
Participant characteristics and adoption of 2023 RIS criteria: **(A)** Geographic distribution of respondents across the DACH region by healthcare setting. **(B)** Number of RIS cases diagnosed in the past 5 years. **(C)** Implementation of 2023 RIS diagnostic criteria stratified by center type; strata are shown only for center types with >5 respondents. Bars in panel **(C)** represent the percentage of centers within each healthcare setting type. The dashed black line indicates the overall percentage across all centers.

A total of 127 neurologists from Germany (79.4%), Austria (17.5%), and Switzerland (3.2%) completed the survey (Figure 1A). Respondents represented a broad range of healthcare settings, including private practices (*n* = 56, 44.1%), university hospitals (*n* = 32, 25.2%), teaching hospitals (*n* = 27, 21.3%), municipal centers (*n* = 6, 5.5%), and other centers (*n* = 5), e.g., rehabilitation centers (3.9%).

Most participants reported diagnosing 5–20 RIS cases over the past 5 years (44.9%), confirming that RIS remains a relatively rare clinical entity (Figure 1B). Higher case numbers (>20) were predominantly reported by university hospitals, where 9.5% had diagnosed more than 50 RIS cases.

### 3.2 Diagnostic approaches

Use of the revised 2023 RIS criteria was heterogeneous. While 33.6% reported consistent use and 41.8% partial implementation, 16.4% had not adopted the criteria, and 8.2% were unaware of these RIS criteria altogether (Figure 1C). This variability was observed across all center types.

**Figure 2:**
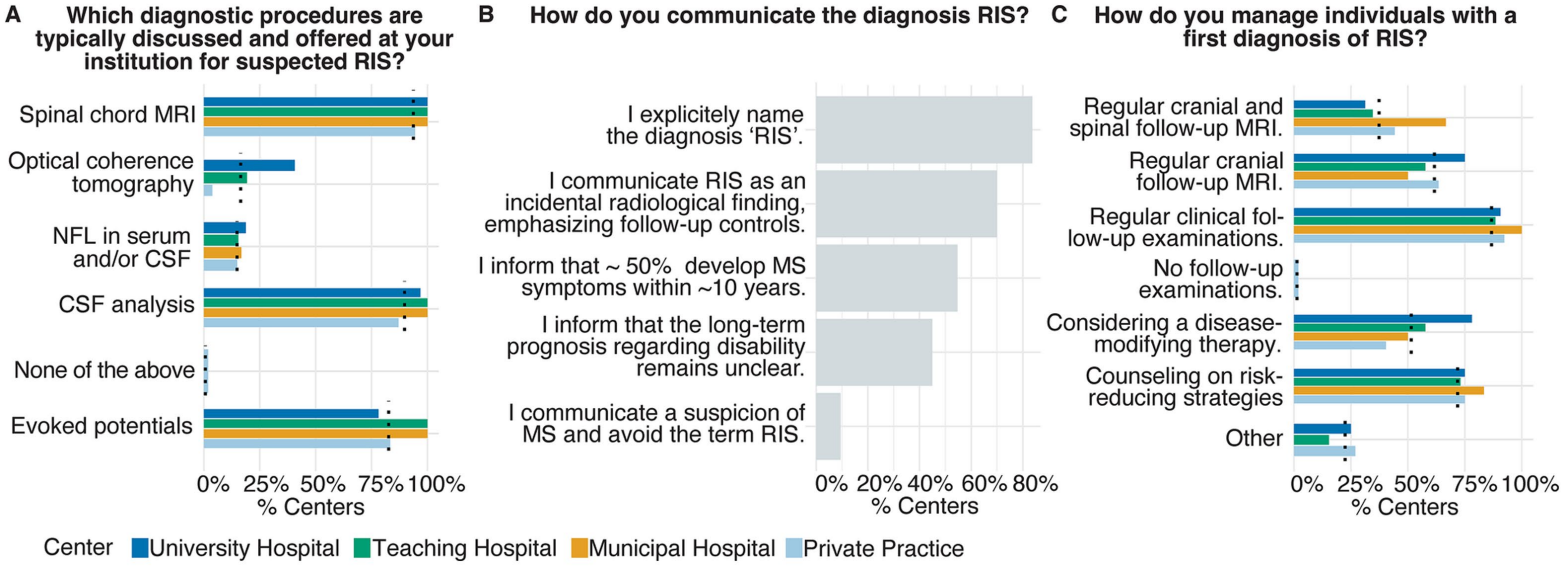
Diagnostic work-up, communication strategies, and management approaches: **(A)** Diagnostic procedures used for RIS evaluation by center types. **(B)** Communication strategies when discussing RIS diagnosis with individuals. **(C)** Management approaches following RIS diagnosis across different center types. Bars in A and C represent the percentage of centers within each healthcare setting type. The dashed black line indicates the overall percentage across all centers. Strata are shown only for center types with >5 respondents.

Despite differing awareness and application of diagnostic criteria, most clinicians reported using a diagnostic work-up for RIS that included spinal cord MRI 97.5%, lumbar puncture 93.4% and evoked potentials 86.1% (Figure 2A). Other biomarkers, such as optical coherence tomography (OCT; 17.2%) or neurofilament light chains analysis (15.6%), were used less frequently and showed substantial variation across centers.

### 3.3 Communication practices and outpatient management

Most clinicians explicitly communicated the term “RIS” (86.8%) and described the finding as requiring monitoring (70%; Figure 2B). The majority (56.2%) informed individuals with RIS about an approximate 50% 10-year risk of developing MS symptoms within 10 years. A minority (9.9%) avoided the term RIS and instead framed the diagnosis as suspected MS.

Similar to the main initial work-up, the management following RIS diagnosis was largely consistent across all settings (Figure 2C). Clinicians reported regular clinical follow-up (91.7%) and lifestyle/risk-factor counseling (75.8%). A total of 65% reported regular cranial imaging, with a preference for annual MRI surveillance (66.7%) compared to 6-month surveillance (20.8%). Both, cranial and spinal MRI monitoring, was more often considered in private practices (44.2%) compared to university hospitals (31.2%). Longer intervals or absence of imaging surveillance were rare.

### 3.4 Treatment approaches and landscape

Regarding treatment initiation following RIS diagnosis, 76.9% of participants discussed evidence from early treatment trials in delaying MS symptom onset with the patient, and 69.4% addressed a lack of approved therapies. Immediate treatment was more commonly considered in university hospitals (78.1%) than in municipal (50%) or private (40.4%) practices, showing variation between centers. Overall, 54.2% considered treatment initiation.

**Figure 3:**
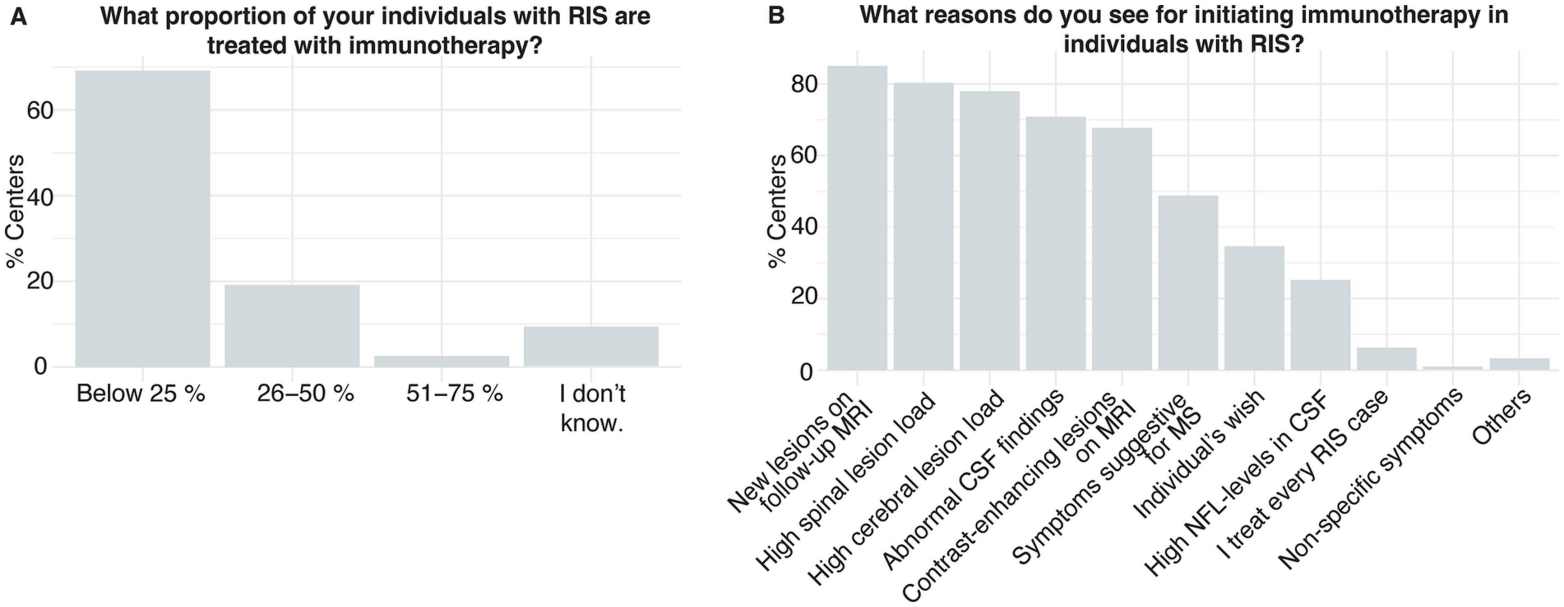
Treatment patterns and triggers for immunotherapy initiation: **(A)** Reported proportion of RIS cases receiving immunotherapy across all centers. **(B)** Clinical and radiological parameters influencing the decision to initiate immunotherapy in RIS.

The proportion of RIS cases receiving therapy differed. Most respondents (69.2%) treat fewer than 25% of their RIS cases (Figure 3A). Only university hospitals reported treating larger proportions (34.3%).

There was broad agreement regarding the reasons for initiating immunotherapy, especially high lesion load in the initial MRI (spinal: 85.0%, cerebral: 82.5%) and the presence of new lesions in the follow-up MRI (90%). For 75% of respondents, an inflammatory CSF syndrome was a reason for treatment initiation, followed by MRI contrast-enhancing lesions (71.7%). Only 6.7% of respondents treat RIS as an early MS by default (Figure 3B).

**Figure 4:**
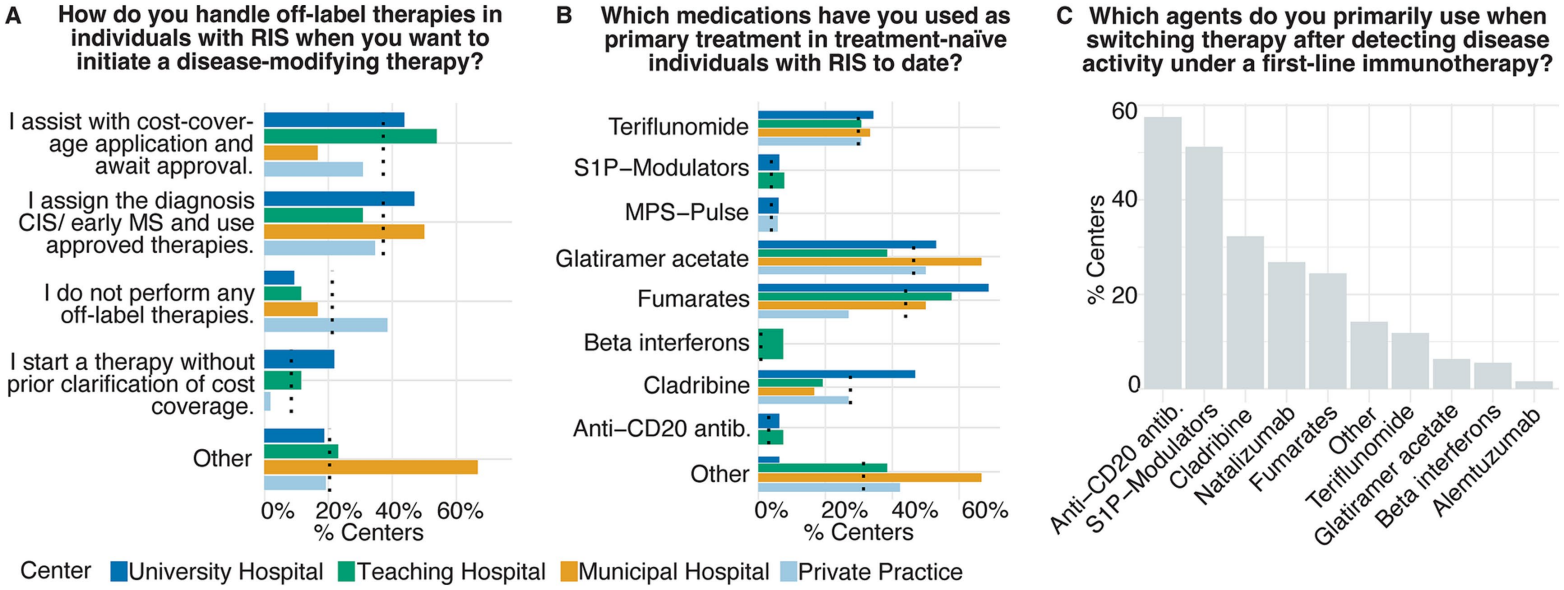
Off-label treatment strategies and therapeutic agents: **(A)** Approaches to handling off-label immunotherapy in RIS and **(B)** first-line disease-modifying therapies used for treatment-naïve RIS patients by healthcare setting. **(C)** Therapeutic agents used when switching therapy due to MRI disease activity. Bars in A and B represent the percentage of centers within each healthcare setting type. The dashed black line indicates the overall percentage across all centers. Strata are shown only for center types with >5 respondents.

To date, no immunotherapy is licensed for RIS. Approaches to off-label treatment varied widely. The two most common strategies were seeking prior insurance approval before starting DMTs (total 39.2%), favored by teaching hospitals (53.8%), or initiating approved DMTs under adapted diagnostic frameworks (total 39.2%), favored by university hospitals (46.9%). Among respondents from private practices, 38.5% reported refraining from off-label treatment. A small fraction of centers (9.2%) reported initiating off-label therapy without prior approval from the insurance provider (Figure 4A).

Initial treatment agents varied, with glatiramer acetate (49.2%) and fumarates (46.7%) being the most frequently employed first-line options overall. Stratified by center type, glatiramer acetate was preferred in municipal hospitals (66.7%), whereas fumarates were preferred in university hospitals (68.8%). Teriflunomide ranked third (30.8%; Figure 4B).

Anti-CD20 agents and S1P-modulators were used rarely across centers (3.3 and 4.1%) for primary treatment intervention but were most used when switching therapy due to disease activity (Figure 4C). This was homogeneous across centers, despite the absence of a standard escalation algorithm for RIS cases reported by most centers (71.7%).

### 3.5 Regulatory and reimbursement barriers: region-independent gaps

Given that RIS is not a distinct ICD diagnosis and that its treatment lacks explicit insurance coverage, we inquired about the key challenges experienced in daily practice. The most frequently cited obstacles were reimbursement challenges for immunotherapies (60.8%) and difficulties with patient education (41.7%). Of note, regional disparities stemming from limited access to neuroimmunology specialists were more commonly reported by neurologists in hospitals (40.7%) than in private practices (5.8%).

Free-text responses revealed four recurring obstacles across all centers: administrative hurdles tied to the uncertainties of ICD coding, the lack of licensed therapies, and, together with the absence of national guidance, these factors cause reimbursement challenges for RIS diagnostics (MRI, spinal tab) and off-label treatment.

### 3.6 Free-text comments

Free-text responses were provided across six open-ended questions addressing key challenges in RIS care. Response rates ranged from 72.5 to 81.7% (*n* = 87–98 respondents per question), with highest engagement observed for questions regarding current gaps in care (*n* = 98, 81.7%) and harmonization needs (*n* = 96, 80.0%).

#### 3.6.1 Impact of recent RIS treatment trials (TERIS and ARISE)

Among respondents addressing the impact of recent treatment trials (*n* = 87, 72.5%), clinicians viewed the TERIS and ARISE trials as providing meaningful therapeutic evidence, thereby increasing their confidence in early treatment intervention for high-risk RIS. Across center types, respondents reported that these studies stimulated more active discussion about immunotherapy and contributed to a sense of emerging therapeutic security. University hospitals (*n* = 23) and teaching hospitals (*n* = 18) respondents reported using these trials to justify off-label treatment more frequently, whereas private practices (*n* = 38) and municipal hospitals (*n* = 8) respondents tended to use trial results primarily to inform patient counseling and individual decision-making.

#### 3.6.2 Perceived changes following introduction of the 2023 RIS criteria

Clinicians responding to questions about the 2023 RIS criteria (*n* = 88, 73.3%) indicated that the 2023 criteria facilitated faster and more confident diagnostic classification by improving the identification of high-risk individuals. Respondents reported greater diagnostic clarity and increased certainty in therapy discussions, alongside a general rise in awareness of RIS within their institutions. Many centers pointed out that, with new criteria emerging, coordinated knowledge transfer and interdisciplinary dialogue have become increasingly important to ensure uniform understanding and application.

#### 3.6.3 Current gaps and inconsistencies in RIS care

When asked about current gaps in RIS care (*n* = 98, 81.7% response rate), respondents identified multiple challenges, including lack of approved therapies, inconsistent diagnostic approaches, heterogeneous imaging quality, and uncertain reimbursement pathways. Private practice respondents particularly emphasized challenges with cost coverage and off-label therapy approval, while university hospitals and teaching hospitals more frequently highlighted the need for standardized diagnostic workflows and imaging protocols.

#### 3.6.4 Perspectives on reimbursement models to support harmonized care

Clinicians responding to reimbursement questions (*n* = 95, 79.2%) expressed that reimbursement frameworks should be aligned with MS care, particularly for diagnostics and monitoring procedures used in RIS. Respondents called for simplified cost coverage for imaging, lumbar puncture, and risk-stratification assessments, as well as the integration of RIS-related services into existing reimbursement MS catalogs. Differences emerged across settings: university hospitals (*n* = 25) and teaching hospitals (*n* = 19) requested documentation-based models that reflected long-term benefits, while private practices (*n* = 42) and municipal hospitals (*n* = 6) highlighted the need for lean reimbursement structures with less paperwork.

#### 3.6.5 Necessity to harmonize RIS care structures

Free-text responses addressing harmonization needs (*n* = 96, 80.0%) highlighted the necessity for national standards for diagnosis and therapy, as well as clearer guidelines for patient counseling. Respondents across all center types emphasized that better data, stronger evidence, and coordinated research efforts are required to anchor treatment decisions and reduce current uncertainty. In addition, participants identified improved knowledge transfer across centers, between radiology and neurology, and within interdisciplinary case conferences as a critical prerequisite for harmonized care. Clinicians consistently pointed to several structural needs for improving RIS care: clearer reimbursement processes, stronger care networks, standardized ICD coding, national imaging standards, and better communication and coordination across institutions.

#### 3.6.6 Role of national recommendations in standardizing RIS care

Participants responding to questions about national recommendations (*n* = 96, 80.0%) consistently emphasized that regional recommendations would offer essential orientation for clinicians, covering diagnostic workflows, therapy approaches, follow-up schedules, and communication standards. Respondents agreed that national guidance would improve legal certainty, support reimbursement negotiations, and reduce structural heterogeneity. While teaching hospitals (*n* = 45) and university centers (*n* = 25) highlighted the need for such guidelines to harmonize care across care levels, private practices (*n* = 43) and municipal hospitals (*n* = 5) stressed the importance of using national recommendations to facilitate reimbursement and reduce administrative burdens. Across center types, clinicians explicitly called for structured platforms to facilitate knowledge transfer and cross-center collaboration, thereby harmonizing RIS care across healthcare sectors.

## 4 Discussion

In this large DACH-wide survey of neurologists conducted in 2024, we identified a combination of heterogeneity and convergence in the real-world management of RIS. While approaches to diagnosis, monitoring, and treatment varied, reflecting the absence of formal guidelines and approved therapies, consistent patterns emerged across healthcare systems and sectors. These findings highlight substantial unmet clinical needs but also reveal areas of shared practices that can serve as a foundation for informed recommendations.

The 2023 RIS criteria broaden the diagnostic window by allowing earlier identification of individuals at increased risk for MS (2). Yet, their adoption remains inconsistent across centers. Nearly one in four neurologists has either not implemented or is unaware of the criteria, indicating a substantial knowledge-to-practice gap. However, given the voluntary nature of this survey, respondents likely represent neurologists with greater interest in and awareness of RIS. The actual knowledge gap among the broader neurological community may therefore be considerably larger, and implementation rates may be lower than reported here. Despite this, most clinicians already perform comprehensive diagnostic workups, aligned with MS diagnostic standards, including spinal cord MRI, CSF analysis, and evoked potentials. This demonstrates that clinical practice in diagnostics is fairly standardized already, despite the absence of formal RIS-specific recommendations.

Management approaches diverged most prominently with respect to structural and systemic constraints. Reimbursement issues, lack of approved therapies, and inconsistencies in national billing systems disproportionately affected private practices and municipal hospitals. Despite these differences, follow-up routines were relatively uniform, with most clinicians conducting regular clinical visits and annual MRI surveillance. This pattern underscores that clinicians share an underlying philosophy of close monitoring but differ in their ability to provide consistent, resource-intensive care. Harmonized guidelines could reduce inequities by defining minimum diagnostic and follow-up standards applicable across all care settings.

Therapeutic decision-making was the most heterogeneous aspect of RIS management. In the absence of approved treatments, clinicians rely on various off-label strategies shaped by institutional risk tolerance and reimbursement feasibility. This led to profound variation in whether and how individuals receive immunotherapy. At the same time, the criteria used to justify treatment initiation were consistent: new MRI activity, high lesion burden (cerebral or spinal), gadolinium enhancement, and inflammatory CSF findings. This shared recognition of biological high-risk constellations aligns with established MS paradigms. The fact that more than 70% of respondents reported a lack of standardized escalation algorithms further underscores the need for defined treatment pathways that guide decisions regarding the initiation and intensification of therapy.

Communication practices showed broad agreement: most clinicians explicitly named the diagnosis “RIS,” communicated an approximate 10-year conversion risk of 50%, and explained both the absence of approved treatments and the emerging evidence supporting early treatment intervention. Yet, the framing of RIS, ranging from an incidental radiological finding to a preclinical stage of MS, varied considerably, influencing patient understanding, emotional burden, and risk awareness. Clear communication standards may help ensure that information on risk, prognosis, and therapeutic uncertainty is conveyed consistently and transparently.

Across all DACH countries and care settings, clinicians reported similar structural challenges: uncertainty about reimbursement for diagnostic procedures, difficulties obtaining approval and coverage for off-label treatments, and the uncertainty of RIS-specific ICD coding linked to defined services eligible for reimbursement. These challenges persist despite differing national healthcare financing systems, indicating that the barriers are driven by overarching conceptual and regulatory factors. A coordinated DACH guideline could provide a unified framework for policymakers and insurers. Concrete steps toward such harmonization should, in our view, include a coordinated DACH-wide consensus statement that defines minimum standards for the diagnostic work-up and monitoring, as well as practical decision aid for the stratification of individuals into low- and high-risk groups to guide treatment decisions, recommendations for patient communication, and dedicated educational programs for caregivers and patients to close the observed knowledge-to-practice gap. In parallel, RIS/preclinical-MS-related healthcare services should be integrated into the existing MS reimbursement and ICD-coding frameworks, so that access becomes more equitable across countries and care sectors.

The free-text responses reinforced these findings and highlighted the importance of recent interventional trials (TERIS and ARISE), which increased clinicians’ confidence in early treatment approaches for high-risk cases and stimulated more active discussion of immunotherapy.(5, 6) University centers more frequently used trial data to justify off-label therapy, whereas private practices and municipal hospitals primarily used them to support structured patient counseling. The 2023 RIS criteria were also viewed positively, as they enable earlier and more reliable diagnostic classification of individuals at high risk (2). However, many centers emphasized that the introduction of new diagnostic criteria has increased the need for knowledge transfer, cross-institutional coordination, and interdisciplinary communication to ensure consistent implementation. There was broad consensus that reimbursement structures should be more closely aligned with MS care and that RIS-related services should be integrated into existing MS billing catalogs.

The revised MS criteria (2024 McDonald Criteria) have implications for the management of high-risk RIS cases, as a subset will now be defined as preclinical MS and thereby gain access to the full diagnostic and therapeutic MS reimbursement framework (4). This reduces regulatory and reimbursement barriers, enhancing opportunities for early therapeutic intervention. At the same time, uncertainties remain regarding monitoring strategies, indicators for treatment initiation, appropriate treatment duration, and communication of the preclinical disease stage. These persistent challenges underscore the need for harmonized structures for RIS and preclinical MS. Standardized diagnostic workflows, uniform monitoring recommendations, treatment pathways, and mechanisms for cross-sector coordination are required to reduce structural heterogeneity in care. Overall, the new MS criteria create a more favorable foundation for managing high-risk individuals, but simultaneously accentuate the need for complementary, DACH-wide recommendations specifically addressing RIS and preclinical MS.

In summary, the survey’s results of marked heterogeneity, persistent uncertainty, and areas of emerging consensus in current practice provide valuable insight into real-world RIS management. While diagnostic and monitoring strategies appear largely aligned and feasible to implement, therapeutic decisions remain highly variable, underscoring the need for clearer treatment frameworks. Evidence from early interventional trials further supports the development of risk-adapted diagnostic and therapeutic pathways. Together, the identified gaps and shared practices form a solid foundation for future guideline updates and the establishment of standardized recommendations for RIS and preclinical MS.

## Data Availability

The raw data supporting the conclusions of this article will be made available by the authors, without undue reservation.
